# Cancer of unknown primary with *EGFR* mutation successfully treated with targeted therapy directed by clinical next-generation sequencing: a case report

**DOI:** 10.1186/s12885-020-07640-4

**Published:** 2020-12-02

**Authors:** Yosuke Mitani, Masashi Kanai, Tadayuki Kou, Shigeki Kataoka, Keitaro Doi, Junichi Matsubara, Shinya Ohashi, Shigemi Matsumoto, Manabu Muto

**Affiliations:** Department of Therapeutic Oncology, Kyoto University Graduate School of Medicine, 54 Shogoin Kawahara-cho, Kyoto, Sakyo-ku 606-8507 Japan

**Keywords:** Case report, Sequencing, Cancer of unknown primary, *EGFR* mutation

## Abstract

**Background:**

Cancer of unknown primary (CUP) is usually treated with nonselective and empirical chemotherapy; however, its prognosis is generally poor, with a median survival of less than a year. Thus, clinicians eagerly await the development of more effective treatment strategies. In recent years, advances in next-generation sequencing (NGS) have made it possible to analyze comprehensively the genome of individual cancers. NGS has identified many genomic alterations, some of which are potential molecular targets of specific agents. We report a case of CUP that was successfully treated with targeted therapy directed by the genomic data obtained from an NGS-based multiplex assay.

**Case presentation:**

A 52-year-old Asian woman with right hip joint pain underwent fluorodeoxyglucose-positron emission tomography/computed tomography, which showed multiple metastatic tumors in her right hip joint, thyroid gland, lung, and vertebrae. Brain magnetic resonance imaging showed multiple cerebral metastases. Additional tests, including pathology examination and conventional epidermal growth factor receptor (*EGFR*) gene mutation analysis (single-strand conformation polymorphism assay), could not identify the primary origin of the tumors, so the patient was diagnosed with CUP. After empirical chemotherapy for CUP, an NGS-based multiplex assay performed using a resected specimen of thyroid tumor detected the *EGFR* mutation c.2573 T > G p.Leu858Arg (L858R). Her treatment was changed to erlotinib, an *EGFR* tyrosine-kinase inhibiter, which dramatically shrank the tumors and decreased her serum carcinoembryonic antigen level. She achieved long-term disease control and survived for 2 years and 9 months from the first diagnosis.

**Conclusion:**

This case might support the strategy that NGS-based multiplex assays could identify actionable molecular targets for individual patients with CUP.

**Supplementary Information:**

The online version contains supplementary material available at 10.1186/s12885-020-07640-4.

## Background

Cancer of unknown primary site (CUP) accounts for 3–5% of all malignancies. It has a poor prognosis, with approximately 10–20% 1-year survival [1], because it has already spread at the time of first diagnosis. CUP has traditionally been treated with nonselective and empirical chemotherapies; however, their efficacy is limited and the development of more effective treatment strategies is eagerly anticipated [2].

With the recent advance of next-generation sequencing (NGS), it has become feasible in daily clinical practice to select the potentially effective therapy for a patient based on comprehensive genetic analysis of his or her individual cancer [3–5]. Here, we report a case of CUP that was successfully treated with targeted therapy based on a comprehensive analysis of the cancer genome, and discuss the possibility of using an NGS-based multiplex assay to inform CUP treatment.

## Case presentation

**Fig. 1:**
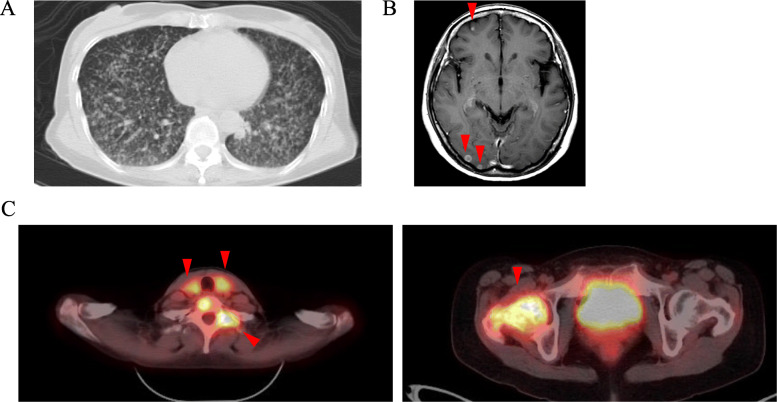
Initial CT, MRI and PET-CT. **a** CT scan showed diffuse pulmonary nodules in both lungs. **b** MRI showed multiple nodules in the cerebral hemispheres. (arrowhead) (**c**) PET-CT demonstrated high FDG uptake in the thyroid, subclavicular lymph nodes, and multiple bones. (arrowhead)

A 52-year-old Asian woman, previously healthy, presented with right hip joint pain. Her medical and family histories were unremarkable. Physical examination revealed painless enlarged lymph nodes in her neck. A full-body computed tomography (CT) scan showed diffuse pulmonary nodules, enlarged thyroid, and osteolytic changes in cervical vertebrae and right femur. Fluorodeoxyglucose-positron emission tomography/computed tomography detected fluorodeoxyglucose uptake in the thyroid, subclavicular lymph nodes, and multiple bones, including the cervical vertebrae and right femur. Brain magnetic resonance imaging showed multiple cerebral tumors (Fig. 1). However, these imaging evaluations plus endoscopy could not identify the primary location of the tumor. Significant laboratory investigations showed an elevated serum alkaline phosphatase (473 U/L; normal range: 115–359 U/L), anti-thyroglobulin antibody (77.8 IU/mL; normal range < 28.0 IU/mL), soluble interleukin-2 receptor (729 U/mL; normal range 145–519 U/mL), and carcinoembryonic antigen (207.7 ng/mL; normal range < 5.0 ng/mL). Thyroid-stimulating hormone (TSH),thyroglobulin, carbohydrate antigen 19–9 and carcinoma antigen 125 levels were within normal limits.

**Fig. 2:**
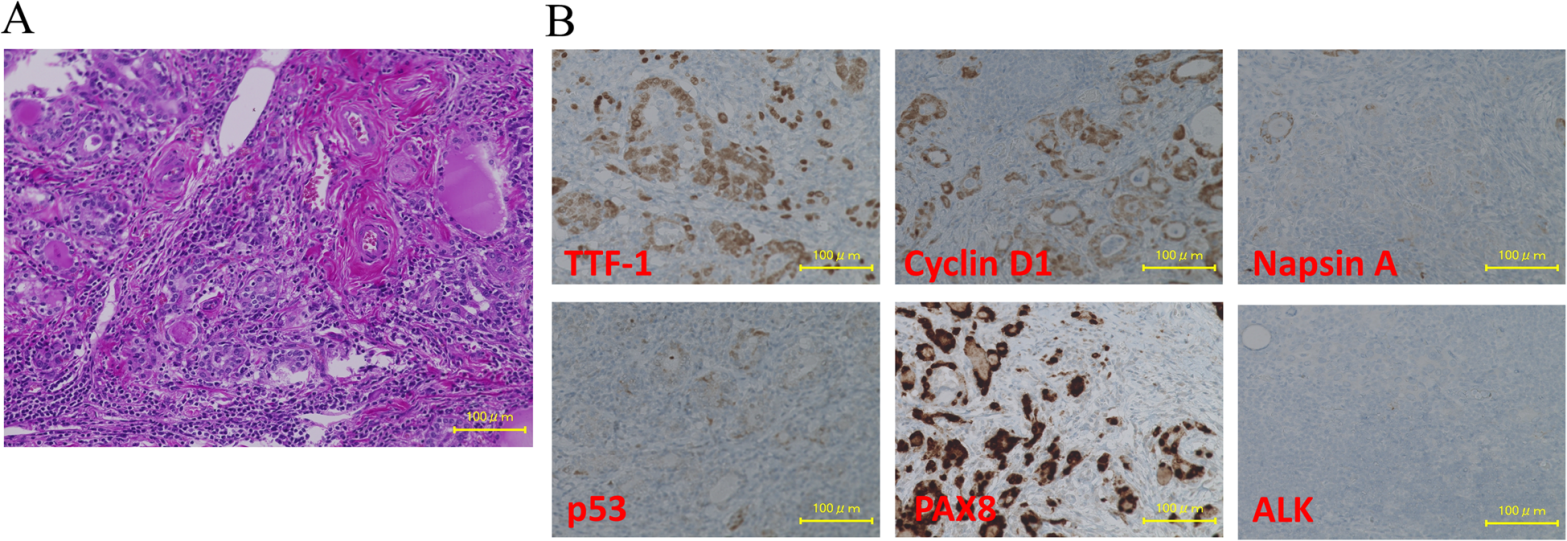
Pathologic findings. **a** H&E Staining of the surgical specimens of total thyroidectomy. **b** On the immunohistochemical examinations the cells were stained positive for thyroid transcription factor 1 (TTF-1), Cyclin D1 and PAX8 and negative for Napsin A, p53 and ALK

Pathological examination of tissue from an ultrasound-guided fine needle aspiration biopsy of the thyroid tumor and the left subclavicular lymph node showed high-grade malignant cells with no clear pattern of differentiation. Because the primary site could not be identified, we decided to treat the patient empirically with intravenous paclitaxel (80 mg/m^2^) plus carboplatin (area under the curve = 2) on days 1, 8, and 15 every 28 days. After 2 cycles of treatment, however, CT evaluation showed an increase in thyroid, lung and bone lesions. Because primary thyroid cancer remained a possibility, we performed a total thyroidectomy with consideration of isotope therapy and reexamination of the pathology. Histopathological examination of the whole thyroid revealed a tumor composed of differentiated cells with glandular lumens and scattered undifferentiated cells (Fig. 2a). On immunohistochemical examination, the cells stained positive for thyroid transcription factor 1, cyclin D1, and paired box 8, and negative for napsin A, *BRAF*(*V600E*), p53, and anaplastic lymphoma kinase (Fig. 2b). These findings suggested adenocarcinoma of thyroid or lung origin, but could not determine the primary location. Epidermal growth factor receptor (*EGFR*) gene mutation analysis using a polymerase chain reaction-single strand conformation polymorphism (PCR–SSCP) assay of the surgical specimen identified no actionable mutation in *EGFR* exon 18, exon 19, or exon 21. Subsequently, the metastatic lesions continued to enlarge. The patient underwent cranial irradiation (30 Gy/10 Fr) and palliative radiation therapy (30 Gy/10 Fr) for painful bone metastases in her cervical vertebrae.

**Fig. 3:**
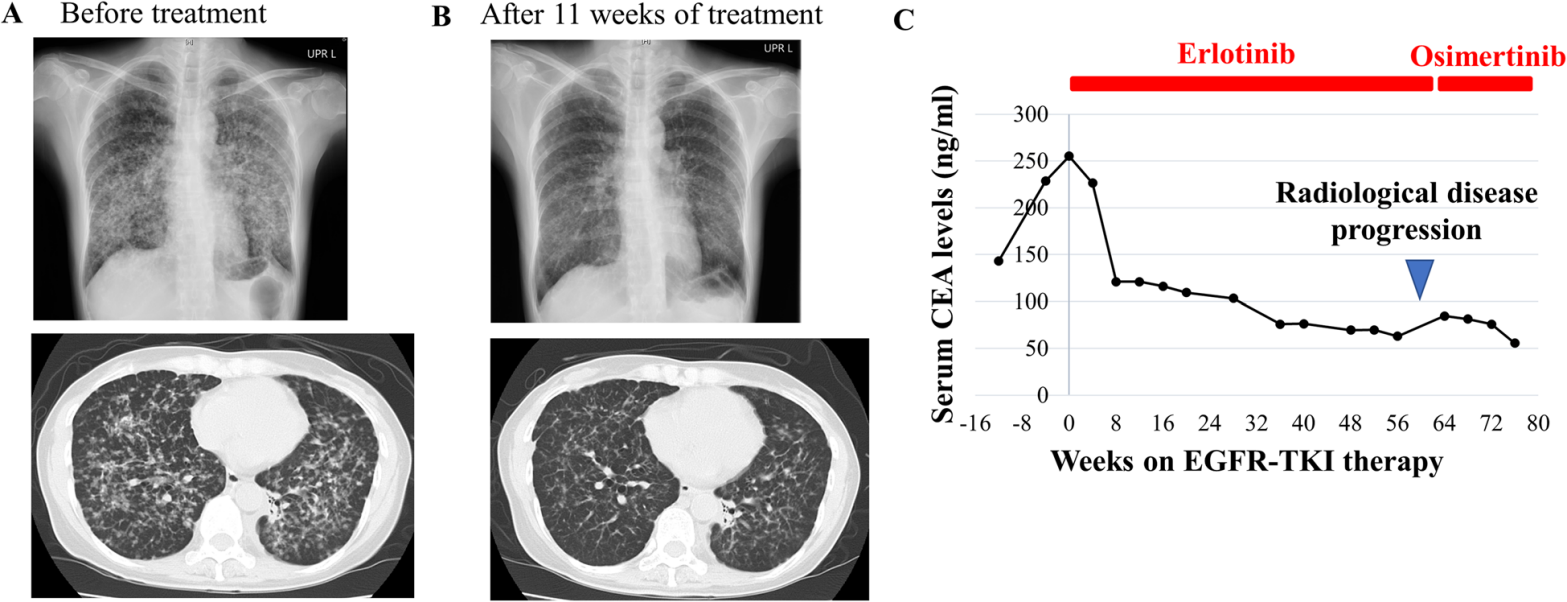
Clinical course after initiation of EGFR-TKI treatment. **a**, **b** A chest X-ray and computed tomography before EGFR-TKI treatment and after 11 weeks. **c** Treatment progress and changes in serum CEA levels

Comprehensive genomic analysis using an NGS-based multiplex assay (Pan-cancer somatic panel PCSP-5.0. R0, Lifecode Health), which was designed to detect variants in 53 cancer-related genes (Supplementary Table 1) and certified concordant with the Clinical Laboratory Improvement Amendments, was performed on a formalin-fixed paraffin-embedded surgical sample of the patient’s thyroid tumor. This assay demonstrated the presence of an *EGFR* mutation, c.2573 T > G p.Leu858Arg (L858R). This mutation is well known to be a positive biomarker for *EGFR* tyrosine-kinase inhibitors (TKI) in non-small-cell lung carcinoma (NSCLC) [6, 7]. Because no standard treatment remained for this patient, we selected treatment with an EGFR-TKI, erlotinib, at 150 mg orally once a day, based on molecular profiling of the tumor. After the first 4 weeks of therapy, the lung lesions showed marked improvement on CT scan and the serum carcinoembryonic antigen level had decreased (Figs. 2 and 3). Fifteen months after the initiation of erlotinib, the patient’s disease progressed with elevation of serum carcinoembryonic antigen level and new cerebral metastasis. Therefore, a liquid biopsy using cell-free circulating tumor DNA was performed for assessment of *EGFR* mutation status, because there were no metastatic lesions accessible for rebiopsy. This analysis identified an additional *EGFR* mutation, c.2369C > T p.Thr790Met (T790M), as well as L858R. The T790M mutation is known to be a cause of acquired resistance to EGFR TKI. However, osimertinib, a third-generation EGFR TKI, is effective for patients with T790M-positive NSCLC, including those with central nervous system metastases [8]. Our patient received osimertinib at 80 mg orally once a day, and her serum carcinoembryonic antigen level decreased again (Fig. 3). She continued the EGFR-targeted therapy and survived for 2 years and 9 months after the first diagnosis, but died because of a general deterioration in her condition that was likely caused by leukoencephalopathy associated with late adverse events of radiotherapy.

## Discussion and conclusion

This patient suffered from CUP with metastasis to multiple organs, including lungs, bones, and cerebrum, which indicates a poor prognosis [9, 10]. Despite these poor characteristics at baseline, she survived for 2 years and 9 months after the initiation of targeted therapy directed by comprehensive genomic profiling.

Her surgical specimen first underwent conventional *EGFR* PCR-SSCP assay, which failed to detect any *EGFR* mutations. We analyzed the same specimen using an NGS-based multiplex assay and identified the actionable mutation of *EGFR*, L858R. The limit of detection for a mutant allele frequency in the *EGFR* PCR-SSCP assay is 10% [11], while in the NGS assay, it is 2–8% [12, 13]. In the present case, the tissue specimen contained plentiful nontumor stromal tissue; thus, the allele frequency might have been below the sensitivity of the *EGFR* PCR-SSCP analysis. This indicates that NGS has great potential as a molecular diagnostic assay in the clinical setting.

The *EGFR* mutation L858R has been reported as a driver mutation in NSCLC [6]. Because this mutation is most commonly observed in lung cancer [6, 14] and is extremely rare in thyroid cancer [15, 16], we speculate that the primary lesion of this patient is in the lung rather than the thyroid. In NSCLC, *EGFR* mutations are strong predictors of efficacy for the EGFR TKIs, and patients whose tumors harbor *EGFR* mutations display a remarkable response rate in prospective trials of TKIs, including randomized phase III trials [7, 17]. However, CUP is not commonly treated with targeted cancer therapy and has historically been treated with empirical therapies using cytotoxic agents. Phase II studies evaluating empirical therapy of CUP have shown a response rate of 23–38% and a median survival of 6–13 months [18, 19]. Thus, comprehensive genomic analysis using a multiplex NGS assay could be helpful to diagnose and treat patients with CUP [2, 4, 20]. For example, in a recent study of 200 patients with CUP, multiplex NGS assay identified one or more potentially targetable genomic alterations in 85% of CUP specimens [21]. In another study of 150 CUP patients, 30% of patients had potentially targetable genomic alterations identified by tumor molecular profiling, and 10% were appropriate for targeted therapies [22]. These studies suggested that a multiplex NGS assay could provide an opportunity for CUP patients to benefit from new personalized therapies.

In summary, we present a patient with CUP who was successfully treated by targeted therapy based on the results of a multiplex NGS assay. This case supports the routine clinical utility of multiplex NGS assays in patients with CUP. We consider that accumulating clinical data about cases of CUP successfully treated after a multiplex NGS assay is relevant for improving the poor prognosis of CUP.

## Supplementary Information

**Additional file 1:** **Supplementary Table 1.** Target genes of the comprehensive genomic analysis.

## Data Availability

All data generated or analyzed during this study are included in this published article.

## Abbreviations

EGFR: Epidermal growth factor receptor
CUP: Cancer of unknown primary
NGS: Next- generation sequencing
CT: Computed tomography
PCR-SSCP: Polymerase chain reaction-single strand conformation polymorphism
TKI: Tyrosine-kinase inhibitor
